# A Microfluidic Prototype System towards Microalgae Cell Separation, Treatment and Viability Characterization

**DOI:** 10.3390/s19224940

**Published:** 2019-11-13

**Authors:** Yanjuan Wang, Junsheng Wang, Chen Zhou, Gege Ding, Mengmeng Chen, Jiang Zou, Ge Wang, Yuejun Kang, Xinxiang Pan

**Affiliations:** 1 Center of Microfluidic and Optoelectronic Sensing, Dalian Maritime University, Dalian 116026, China; wangyanjuan@dlmu.edu.cn (Y.W.); zhouccc@dlmu.edu.cn (C.Z.); dgglock@dlmu.edu.cn (G.D.); cmm13204069538@dlmu.edu.cn (M.C.); a1137587987@dlmu.edu.cn (J.Z.); wyh2019@dlmu.edu.cn (G.W.); 2 College of Information Science and Technology, Dalian Maritime University, Dalian 116026, China; 3 Software Technology Institute, Dalian Jiaotong University, Dalian 116028, China; 4 Navigation College, Guangdong Ocean University, Zhanjiang 524088, China; 5 School of Materials and Energy, Southwest University, Chongqing 400715, China; yjkang@swu.edu.cn; 6 College of Electronics and Information Engineering, Guangdong Ocean University, Zhanjiang 524088, China; panxx@dlmu.edu.cn

**Keywords:** microfluidic chip, deterministic lateral displacement, concentration gradient generator, single photon detection, chlorophyll fluorescence

## Abstract

There are a huge number, and abundant types, of microalgae in the ocean; and most of them have various values in many fields, such as food, medicine, energy, feed, etc. Therefore, how to identify and separation of microalgae cells quickly and effectively is a prerequisite for the microalgae research and utilization. Herein, we propose a microfluidic system that comprised microalgae cell separation, treatment and viability characterization. Specifically, the microfluidic separation function is based on the principle of deterministic lateral displacement (DLD), which can separate various microalgae species rapidly by their different sizes. Moreover, a concentration gradient generator is designed in this system to automatically produce gradient concentrations of chemical reagents to optimize the chemical treatment of samples. Finally, a single photon counter was used to evaluate the viability of treated microalgae based on laser-induced fluorescence from the intracellular chlorophyll of microalgae. To the best of our knowledge, this is the first laboratory prototype system combining DLD separation, concentration gradient generator and chlorophyll fluorescence detection technology for fast analysis and treatment of microalgae using marine samples. This study may inspire other novel applications of micro-analytical devices for utilization of microalgae resources, marine ecological environment protection and ship ballast water management.

## 1. Introduction

Microalgae are among the simplest and oldest microorganisms on the earth, which have diverse populations and widely distributed with footprints from tropic to polar areas. It is estimated that there are more than 50,000 species of microalgae on the planet and more than 30,000 species have been discovered. Microalgae are one of the most important components of the marine food chain and primary producers in marine ecosystems [1]. Most microalgae have thylakoids, which allow photosynthesis by absorbing CO_2_ and producing oxygen, and thereby reduce the greenhouse effect. Marine microalgae can synthesize high-quality proteins using inorganic carbon and carbohydrates, and regenerate bio-metabolites in the ocean into valuable sources for other organisms. In addition, microalgae also have important economic values for humanity because they provide rich nutrients and bioactive reagents, including proteins, polyunsaturated fatty acids (PUFA), polysaccharides, biological antibiotics, carotene, astaxanthin, phycocyanin, multivitamins, oils and other trace elements. Therefore, microalgae have found broad applications in many critical fields, such as food, healthcare, energy and environment [2,3,4].

Applications of microalgae are dependent on the specific properties of different species. For example, *Anacystis* and *Symechococcus* are common model microorganisms for the study of molecular genetics. *Spirulina* and *Arthrospira* can be used as dietary supplements to enhance the immunity of the body, regulate glucose levels, or control cholesterol levels, thereby reducing the risk of heart diseases or stroke. *Haematococcus* is an important source of astaxanthin. *Chlorella* produces a rich amount of intracellular oil, and thus, has a promising potential as a key source of bioenergy. *Pyramimonas* sp., *Platymonas*, *Chaetoceros*, etc. have high nutritional value and have been usually used as feed [5,6]. Obviously, identification or separation of particular species of microalgae is a prerequisite for the profound investigation of algal biology, and further utilization of microalgae resources [7].

Traditional methods for separation of microalgae mainly depend on microcapillary, dilution plate, waterdrop, antibiotic, or solvent extraction [8,9]. These methods usually require laborious and time-consuming operations in laboratory settings with bulky equipment, for example, centrifugation and filtration. Novel strategies with a compact system, low cost, fast processing speed and convenient operation are urgently needed for separation and rapid analysis of microalgae. To date, microfluidics-based platforms have been extensively used in biomedical, materials engineering and environmental applications to realize sample preparation, reaction, detection, separation and many other tasks on a single chip system [10,11,12]. Microfluidic separation technology can be divided into active separation and passive separation method. Common active separation methods include electrical separation, optical separation, acoustic separation and magnetic separation [13,14]. However, these methods need to add externally applied force fields to achieve separation, and the device is large in size, which is not conducive to integration. The passive separation method mainly relies on the hydrodynamics, as well as the size and flexibility of the cells to achieve separation. For example, microstructure filtration, inertial separation, and DLD separation techniques. These methods do not require external equipment, and are easy to achieve miniaturization, portability and integration. Microstructure separation is achieved by designing different grooves, herringbones or microwells in the microchannels, and separated according to cell size, density, or deformability, etc. [15]. This method is simple to operate, but prone to clogging. Moreover, excessive extrusion force may cause damage to the cells. Inertial separation is a simple and high-throughput separation method [16,17]. Different sizes of particles have different equilibrium positions in the inertial channel, but the separation efficiency is relatively low. DLD technology achieves separation by designing micro-pillar arrays of different sizes and shapes in microchannels, which has unique advantages in terms of processing speed, throughput, label-free and convenience for integration [18,19,20,21,22]. It is promising for applications in microalgae separation.

Although microalgae are a class of critical and valuable natural resource, they could bring serious environmental issues, e.g., red tides, if out of control. Red tide is caused by human activities, nutrient enrichment in water, etc., causing a sharp increase in biomass, such as microalgae, leading to the destruction of ecological balance and deterioration of water quality [23,24]. The massive discharge of ship’s ballast water without effective inactivation treatment is one of the important causes of red tides [25]. With the thriving development of marine transportation, a tremendous amount of ballast water is produced annually. There are several billions of microorganisms contained in every cubic meter of ballast water, most of which are microalgae. If the massive number of microalgae in ballast water are not properly controlled, they may cause a disastrous effect on the stability of the local marine ecosystem, diversity of biological species and even human health [26,27,28,29]. Therefore, detection and inactivation of microalgae is an important field for red tide management and marine environment protection.

Conventional methods to inactivate water-borne microorganisms mainly include ultraviolet irradiation, electrolysis, ozone, chemical reagent. The traditional methods eradicate all the creatures in the water without distinction. If the samples were separated before inactivation, the inactivation characteristics of each species could be studied, so as to select a more suitable inactivation method, as well as the appropriate reagent and concentration. Chlorination is a commonly used method of inactivation in chemical processes. It is dependent on the hydrolyzed NaClO to destroy cell wall and cytomembrane of microorganisms, and has proved to be a highly effective treatment to various microorganisms [30,31,32]. However, the application of chemical reagents needs to be carefully controlled, because the insufficient dosage cannot completely inactivate the target microorganisms, while excessive chemical residues may cause secondary pollution to the marine ecological environment. Therefore, it is important to optimize the concentration and processing time of the chemical reagent during the treatment. For this purpose, the viability of microalgae is usually analyzed as a direct indicator of the treatment effect. Traditional methods to evaluate microalgae viability rely on microscopic observation, which is time-consuming, laborious and introduces many human errors. It has been reported that chlorophyll of microalgae emits laser-induced fluorescence, and the intensity of fluorescence is proportional to the amount of chlorophyll in microalgae [33]. Therefore, the viability of the microalgae can be more accurately characterized by detecting the intensity of induced fluorescence.

Based on the above analysis, herein, we proposed a microfluidics-based analytical system that comprises microalgae separation, inactivation and viability analysis. Specifically, rapid separation of microalgae by size was realized based on the DLD principle. Through separation, the inactivation characteristics of different species of microalgae cells can be studied, which is the prerequisite for selecting the most suitable inactivation method. Additionally, a microfluidic concentration gradient generator was designed to optimize the concentrations of treatment reagent for effective inactivation of target microalgae, which avoids the cumbersome and error of manual operation. Finally, a single photon detection system was used to accurately evaluate the viability of microalgae cells based on the laser-induced fluorescence of intracellular chlorophyll. Compared to the traditional methods, this new analytical system is very compact, much less costly, with fast processing speed. In this study, two species of microalgae (*Pyramimonas* sp. and *Chlorella*) were adopted to demonstrate the performance of the proposed microfluidic system.

## 2. Materials and Methods

### 2.1. Theories

#### 2.1.1. Theoretical Analysis of DLD

**Figure 1:**
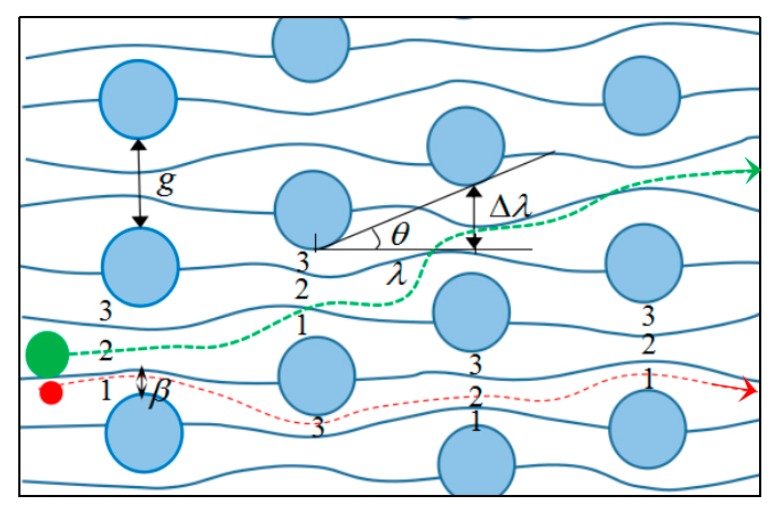
Principle of the deterministic lateral displacement.

DLD is a size-based continuous separation method using an asymmetric array of micro-posts, where separation is achieved by the special flow characteristics at low Reynolds numbers and the interaction of the particles with the posts. The trajectory of the particles is predictable, theoretically. Particles below a critical size (diameter, *D_C_*, for spherical particles) travel along with the global flow, whereas, particles above this size undergo a lateral drift. The micro-posts may have various shapes, such as triangles and trapezoids. In the present study, an array of circular posts (Figure 1) were used because they are easy to fabricate and relatively stable. The curves between the posts represent the flow streamlines, and *β* represents the width of the first streamline. If the particle radius is smaller than *β*, it will move along the original streamline in a zigzag course (red line in Figure 1). If the particle radius is larger than *β*, there will be a lateral drift when it moves on (green line in Figure 1) [34,35]. Therefore, the width of the first streamline *β* is a key factor to determine the particle trajectory, and the relationship between *β* and critical size *D_C_* is denoted below,

(1) $$D_{C}=2\beta .$$

The key to achieving particle separation by DLD is to design a chip containing micro-post arrays with suitable critical particle size *D_c_*. As shown in Figure 1, *θ* is the tilt angle between two adjacent rows of posts, *λ* represents the horizontal distance between two adjacent rows of posts, and Δ*λ* represents the vertical shift distance between two adjacent rows of posts. The row shift fraction *ε* is defined as the ratio of the vertical shift Δ *λ* to the horizontal distance *λ*,

(2) $$\varepsilon =\frac{\Delta \lambda }{\lambda }=\tan\theta .$$

The relationship between the vertical spacing *g* and the flow velocity *v* between the posts is represented by *μ* (*x*), where *x* represents the distance from the edge of a micro-post. The velocity is zero at the edge of the post according to the non-slip boundary condition. The velocity field around the circular posts is symmetrically distributed and can be represented by parabolic functions. Three typical boundary conditions of the parabolic functions are (0, 0), (*g*, 0), (*g*/2, *v*_max_), which can be substituted in a parabolic function yielding,

(3) $$\mu (x)=-\frac{4v_{\max}}{g^{2}}x^{2}+\frac{4v_{\max}}{g}x.$$

The ratio between fluid flux across the first streamline width *β* and the total flux across the vertical spacing *g* is equal to the row shift fraction *ε* [36],

(4) $$\int_{0}^{\beta }\mu (x)dx=\varepsilon \int_{0}^{g}\mu (x)dx.$$

Substituting Equations (1) and (3) into Equation (4) and solving for *D_c_*,

(5) $$D_{c}=g\left[1+2M+\frac{1}{2M}\right].$$

where *M* represents,

(6) $$M^{}={\left[\frac{1}{8}-\frac{\varepsilon }{4}+\sqrt{\frac{\varepsilon }{16}(\varepsilon -1)}\right]}^{\left(\frac{1}{3}\right)}\left(-\frac{1}{2}-i\frac{\sqrt{3}}{2}\right).$$

Therefore, the critical particle size, *D_c_*, varies with the vertical spacing *g* and the row shift fraction *ε*. Accordingly, a DLD chip can be designed to satisfy the requirements for separation of target microalgae species with unique sizes.

#### 2.1.2. Theories of Concentration Gradient Generator

In a typical concentration gradient generator, original reagent solution and dilution buffer are injected into different inlets, mixed in proportion at different nodes and bifurcation points, and separated again at the next level. A series of flow splitting and remixing result in automatic and rapid generation of a precisely controlled concentration gradient. In a microfluidic concentration gradient chip, the Reynolds number (Re) of the laminar flow is much less than unity. Assuming the fluid is Newtonian with constant viscosity, the laminar flow in a microfluidic chip satisfies Hagen-Poiseuille’s law [37]:

(7) $$\Delta p=QR_{H}^{},$$

where *Q* is the flow rate, Δ*p* is the pressure difference, and *R*_H_ is the fluid resistance. It is known that when the channel cross-sectional shape is fixed, the flow resistance is proportional to the channel length *L*, which can be used as a designing variable to control the flow resistance.

**Figure 2:**
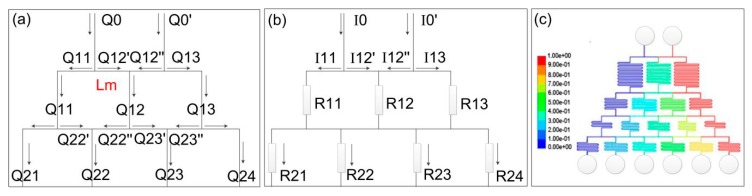
(**a**) The equivalent flow diagram of the concentration gradient generator; (**b**) The equivalent electric circuit corresponding to (**a**); (**c**) The simulated concentration profile in the entire chip.

For the complex flow network in a gradient generator, an equivalent electric circuit system is usually used for the flow analysis based on circuit principle [38,39]. In fact, Equation (7) is analogous to the Ohm’s law governing the electric current analysis, in which the flow resistance *R*_H_ is analogous to the electric resistance *R*, the flow rate *Q* is analogous to the electric current *I*, and the pressure drop Δ*p* is analogous to the electric voltage *V* [40,41]. Figure 2a shows the typical tree-like structure of a microfluidic concentration generator, which is equivalent to the electric circuit in Figure 2b. The microfluidic flow satisfies the Kirchhoff’s current law, denoting the mass conservation at the nodes of flow circuits. More specifically, the sum of input flow rate *Q*_in_ at any node is equal to the sum of output flow rate *Q*_out_, denoted as:

(8) $$\sum_{n=1}^{N}Q_{n}=0.$$

Considering the conservation of reagents in the fluids with different concentrations, the following relationship between concentration *C* and volumetric flow rates *Q* also applies [42]

(9) $$C_{ij}=\frac{{Q_{ij}}^{\prime }}{Q_{ij}}C_{i-1j-1}+\frac{{Q_{ij}}^{\prime \prime }}{Q_{ij}}C_{i-1j},$$

where *i* denotes the *i*-th row of the microfluidic circuit from top to bottom, and *j* denotes the *j*-th branch from left to right.

Before designing the exact structure of the concentration gradient chip, we need to determine the required concentrations and flow rates at each outlet. The two inlet flows are of concentrations of 0% and 100%, representing the diluting buffer and the original reagent solution, respectively. For example, set the concentration of each outlet to 0%, 20%, 40%, 60%, 80%, 100%. The concentrations at each downstream level in the chip can be set as any value lower than those at the upstream levels. Here we assume that the concentration of each level is shown in Table 1:

In the microfluidic chip, the relationship between flow rate and concentration is mutually constrained. According to Equations (8) and (9) and Table 1, the flow rate of each level can be calculated. Assuming the flow rate at each outlet is 2 μL/min, the flow rate at each inlet can be calculated as 6 μL/min. Obviously, the flow resistance of the straight horizontal connection channel (we set L_m_ = 3 mm) is much smaller than the curved mixing channel arranged in the vertical direction. Then, according to Kirchhoff’s voltage law and Equation (7), we can derive the flow resistance at each level of channels:

For *i* = 4 (bottom level):

(10) $$R_{4,j}=\frac{Q_{4,j+1}R_{4,j+1}+{Q_{4,j+1}}^{\prime }R_{m}-{Q_{4,j}}^{\prime \prime }R_{m}}{Q_{4,j}}.$$

For *i* < 4:

(11) $$R_{i,j}=\frac{Q_{i,j+1}R_{i,j+1}+{Q_{i,j+1}}^{\prime }R_{m}+{Q_{i+1,j+1}}^{\prime \prime }R_{m}-{Q_{i,j}}^{\prime \prime }R_{m}-{Q_{i+1,j+1}}^{\prime }R_{m}}{Q_{i,j}}.$$

Because flow resistance is proportional to channel length under the fixed cross-sectional area, the channel length at each level can be calculated according to the concentrations and flow rates at the outlets of each level using Equations (10) and (11).

In this way, we designed a concentration gradient generation chip, as shown in Figure 2c. To mix the liquid thoroughly, the channels in the mixing zone have a serpentine layout. The channel width is 100 μm. The color in Figure 2c denotes the simulated reagent concentrations in the chip, with outlet concentrations of 0%, 20%, 40%, 60%, 80% and 100% from left to right.

### 2.2. Sample Preparation

The microalgae samples, *Pyramimonas* sp. (diameter 10–15 μm) and *Chlorella* (diameter 3–5 μm), were obtained from Liaoning Ocean and Fisheries Science Research Institute (Dalian, Liaoning). Each microalgae species was cultured individually in a conical flask containing an enriched seawater medium, which was shaken gently every three hours. They grew in a CO_2_ incubator under a photoperiod of 12 h. After that, 15 mL of each microalgae species was centrifuged at 8000 rpm for 10 min at room temperature (AllegraTM X-22R Centrifuge, Beckman Coulter, Brea, CA, USA) and the supernatant was removed. Then, the microalgae were resuspended in PBS buffer solution and re-centrifuged twice before analysis. Through centrifugation, most of the impurities can be removed. NaClO solution was used to inactivate microalgae. The concentration of NaClO solution was determined by an iodometric method as described previously [43].

### 2.3. System Design and Operation

**Figure 3:**
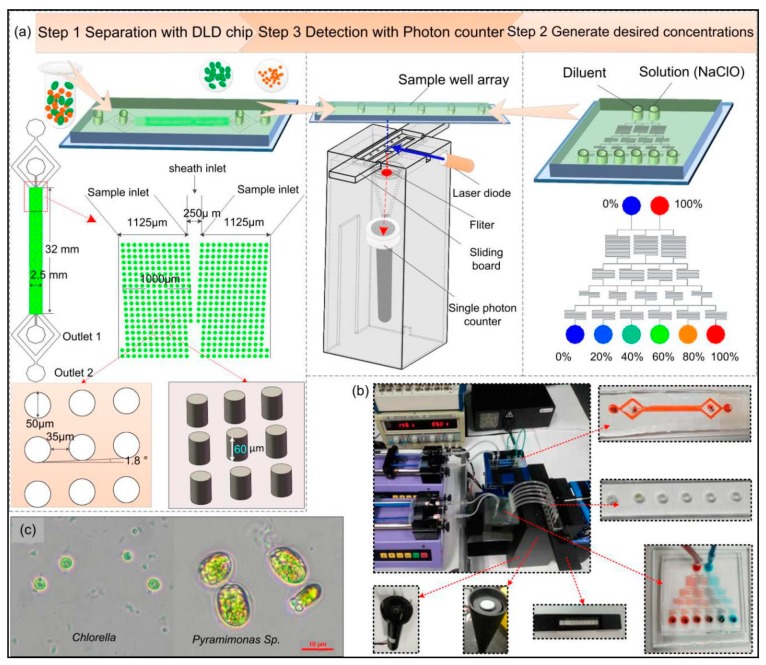
(**a**) The schematic diagram of the designed system. (**b**) The physical map of the system. (**c**) The photomicrograph of *Pyramimonas* sp. And *Chlorella*.

Based on the above analysis, a novel microfluidic system was designed in this study, the schematic diagram of the designed system is shown in Figure 3a, which has three modules, including microalgae separation, treatment and viability analysis. The separation module consists of a DLD separation chip, microscope, and a syringe pump. The DLD separation chip was fabricated in PDMS using a mold of SU-8 fabricated by UV photolithography [44]. In the DLD separation chip, it consists of two inlets and two outlets. The inlet in the center is the sheath flow; the width of the sheath inlet is 250 μm. The side inlet is the sample flow, and it is divided into two branches, the width of each branch is 1125 μm. Two symmetrical micro-posts arrays are designed in the separation region. The width of the micro-posts array on the one side is 1 mm, the overall length of the array is 32 mm. The vertical and horizontal spacing between the micro-posts is designed to be the same, both 35 μm. The tilt angle *θ* is 1.8°, post diameter is 50 μm, channel height is 60 μm, and *D_C_* is 7.5 μm (details of the fabrication process and the calculation are available in the supporting information). The treatment module is mainly composed of a concentration gradient generator and a sample well array. The cross-section of the concentration gradient generation chip is rectangular. The viability analysis module is composed of a single-photon counter, a filter, laser source, data acquisition and display module, and a slide control module. The actual setup of this system is shown in Figure 3b, and Figure 3c shows the photomicrograph of the samples (*Pyramimonas* sp. And *Chlorella*).

The first step of the experiment was to differentiate microalgae according to size using the DLD separation system. Before the experiment, the chips need to be cleaned in a plasma cleaner (Harrick Plasma PDC-32G, Ithaca, NY, USA) for 2 min to render a hydrophilic channel surface. Then, flush the channel with absolute alcohol to further improve the hydrophilicity of the channel. To avoid damage to the cells from residual alcohol, flush the channel with PBS solution for 5 min and ensure that all air bubbles are expelled. After that, a syringe pump (Harvard apparatus Co., Inc., Holliston, MA, USA) was used to inject the samples (the mixture of *Pyramimonas* sp. And *Chlorella*) and sheath fluid (PBS solution) into two entrances of the separation chip at a constant flow rate. Based on DLD principle, *Pyramimonas* sp. cells that are larger than the critical size would undergo a deterministic lateral shift, and flow out from Outlet 1, while *Chlorella* cells that are smaller than the critical size would remain in the original stream, and flow out from Outlet 2, thus, realizing the separation of these two species. The two species of microalgae cells after separation were collected in tubes individually, for further study.

The second step was to quickly and accurately generate the desired concentrations of NaClO solution. Firstly, connected six outlets of the concentration gradient generator with the sample well array using PVC tubes. Then, the diluent (deionized water) and NaClO solution (100%) were respectively pumped into the two inlets of the concentration gradient generation chip at a flow rate of 6 μL/min. The corresponding flow rate at each outlet was 2 μL/min, and the injection was stopped after 5 min. After fully mixing in the chip, NaClO solution with concentrations of 0%, 20%, 40%, 60%, 80% and 100% were obtained. Afterwards, the microalgae cells were uniformly oscillated and manually added to the six sample wells using a pipette.

The final step was an evaluation of the inactivation effect on microalgae under different concentrations of NaClO using a single-photon counter to determine the most appropriate reagent concentration and optimal treatment time. The analysis was based on laser-induced fluorescence of chlorophyll in microalgae, which emits strong fluorescence near the wavelength of 685 nm under the excitation at 488 nm. The viability of microalgae was characterized by the intensity of chlorophyll fluorescence. We used a multi-channel laser source (90-10034, Lumencor, Beaverton, OR, USA) transmitted by optical fibers, and the output wavelength could be adjusted according to specific requirements. To minimize the background signals, we placed a filter (ET670/50M, Chroma ATE Inc., Novi, MI, USA) in front of the receiver, after which the emission light was received by the single-photon counter (H7360-03, Hamamatsu, Bridgewater, NJ, USA). The counter converts the received photons into photoelectrons, which are amplified and converted into pulsed signals at a certain frequency (the number of photoelectron pulses per unit time determines the fluorescence intensity). Then, the output pulsed signals were counted and stored by a NI data acquisition system, and displayed on a computer in real time using LabVIEW software.

## 3. Results and Discussion

### 3.1. Microalgae Separation

#### 3.1.1. Analysis of Microalgae Movement

**Figure 4:**
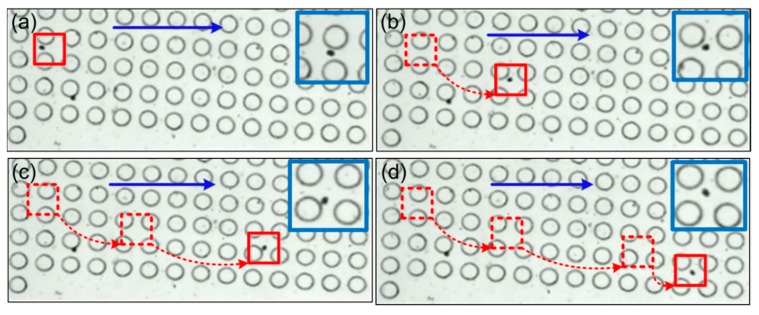
The trajectory of one *Pyramimonas* sp. cell in 4 consecutive frames ((**a**–**d**), and the time differences is 0.04 s). The blue arrows indicate the original direction of the sample flow.

We firstly analyzed the motion trajectory of a single algae species. The sheath and sample flow rates were fixed as 5 μL/min and 15 μL/min, respectively. The purpose of sheath flow is to provide hydrodynamic resistance and prevent small-sized cells from diffusing into the central enrichment zone. Four consecutive frames of a moving *Pyramimonas* sp. Single cell are shown in Figure 4. As discussed above, cells larger than the critical size will undergo lateral displacement after collision with the micro-posts, and deviate from the original flow stream. Because of the size of a single *Pyramimonas* sp. Cell is greater than the critical size of the current micro-post arrangement; and there was an obvious lateral displacement downward to the central enrichment zone, as shown in the deviated trajectory. This finding was in line with the above theoretical analysis of DLD mechanism.

#### 3.1.2. The Effect of Flow Rate on Separation Efficiency

Because DLD separation is mainly based on hydrodynamics and physical properties of particles, the flow rate usually has a notable impact on the separation efficiency. The flow rate effects include two important aspects, including the relative flow rates of sheath and sample, and the overall flow rate on the chip. We will discuss these two aspects individually as below.

**Figure 5:**
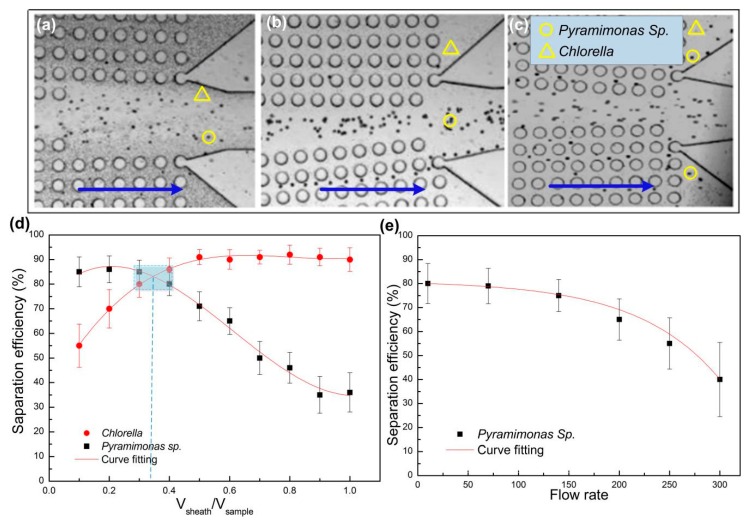
The effect of relative flow rates of sheath and sample flows on separation efficiency. Trajectories of microalgae cells under (**a**) V_sheath_ = 1.5 μL/min, V_sample_ = 15 μL/min; (**b**) V_sheath_ = 5 μL/min, V_sample_ = 15 μL/min; (**c**) V_sheath_ = 10 μL/min, V_sample_ = 15 μL/min; (**d**) The separation efficiencies of both microalgae species under different flow rate ratios V_sheath_/V_sample_; (**e**) The effect of overall flow rate on separation efficiency.

In order to study the effect of relative flow rates of sheath and sample on the separation efficiency, we kept the sample flow rate constant (15 μL/min), while the sheath flow rate was gradually increased from 1.5 to 15 μL/min. As shown in Figure 5a, when the sheath flow rate was much lower than the sample flow rate, *Pyramimonas* sp. cells and *Chlorella* cells both entered the central enrichment zone, indicating insufficient flow resistance provided by the sheath flow to prevent *Chlorella* from entering the enrichment zone. When the sheath fluid increased to 5 μL/min, almost all *Pyramimonas* sp. cells entered the central region, while most *Chlorella* cells flowed into the side channel (Figure 5b). When the sheath flow was further increased to 10 μL/min, due to the high flow resistance of the sheath flow, some of the *Pyramimonas* sp. cells failed to enter the central enrichment area, and flow out from the side channel (Figure 5c). These results revealed that there was an optimal relatively flow rate of sheath flow to the sample flow, which needs to be carefully adjusted to ensure efficient separation of these two microalgae species.

We further evaluated the effect of relative flow rate on the separation efficiency under various flow rate ratios (V_sheath_/V_sample_). A hemocytometer (XB-K-25) was used to count the number of target cells collected in corresponding outlets, which was divided by the total number of target cells collected from all outlets to calculate the separation efficiency (Figure 5d). Obviously, the separation efficiency of *Pyramimonas* sp. cells (in the central enrichment outlet) decreased with the flow rate ratio, while the separation efficiency of *Chlorella* cells (in the side outlet) increased with the flow rate ratio. The flow rate ratio to achieve optimal separation efficiency for both microalgae species was about 1/3 for this chip setting.

The working throughput of the chip is determined by the overall flow rate (Here, we define the sample flow rate as the overall flow rate, while adjusted the sheath fluid flow rate to match the sample flow rate). We increased the overall flow rate in the chip from 10 to 300 μL/min. The results in Figure 5e shows that the separation efficiency of *Pyramimonas sp* began to decrease when the overall flow rate increased to above 70 μL/min, as the sample speed increased to 200 μL/min, the separation efficiency shows a sharp downward trend. This could be due to the increasing flow resistance under very high flow rate that restrained the deterministic lateral displacement of the microalgae cells.

#### 3.1.3. The Separation of Mixed Microalgae

**Figure 6:**
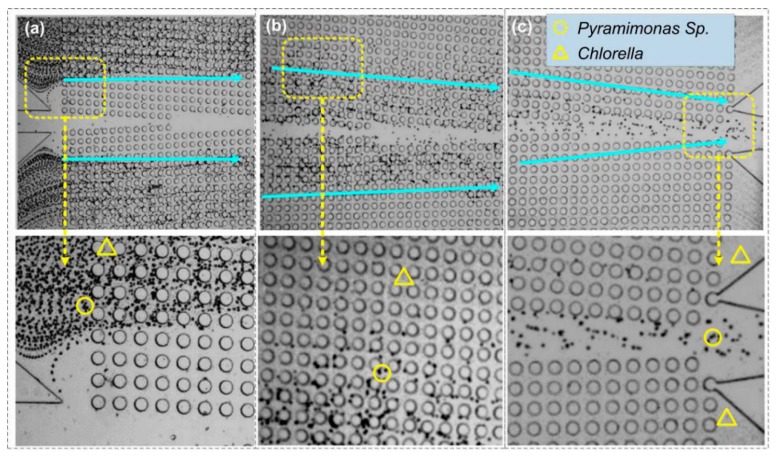
The trajectories of mixed microalgae cells (circles denote *Pyramimonas* sp., triangles denote *Chlorella*) at different locations in the DLD chip: (**a**) Entrance, (**b**) middle section, and (**c**) outlets. The solid arrows indicate the general direction of microalgae movement. The photos below are an enlargement of the dashed box in the corresponding above photos.

We further separated the mixture of these two microalgae species. The sample (15 μL/min) and the sheath flow (5 μL/min) were introduced into the inlets of the DLD separation chip using a syringe pump. Figure 6 showed the trajectories of microalgae at the entrance, middle and outlets of the chip. At the chip entrance, the mixed microalgae streams were squeezed by the central sheath to flow within the micro-posts array (Figure 6a). Notable lateral displacement occurred in the middle section of the chip for *Pyramimonas* sp. cells that collided with the micro-posts. Meanwhile, *Chlorella* cells still remained in their original sample streamline without an obvious lateral shift, as shown in the enlarged view in Figure 6b. Finally, (Figure 6c), almost all *Pyramimonas* sp. cells flowed into the central enrichment zone near the chip outlet. The separation efficiency of the *Pyramimonas* sp. cells collected from the central outlet channel reached more than 80%, while the *Chlorella* cells collected from the side channels exceeded 85%.

### 3.2. Microalgae Inactivation Using Concentration Gradient Generator

After obtaining separated species of microalgae, as shown above, we can further study the inactivation effect on the single target species, the conventional method of inactivating microalgae is usually performed by indiscriminating eradication of all microalgae in the sample, which is of low efficiency and may cause secondary pollution, due to excessive use of chemical reagent. To address this issue, we aim to optimize the concentration of NaClO for inactivating the separated single species of target algae using chlorination method. The chlorination effect is based on the oxidative damages of hydrolyzed NaClO on microalgae cells, which penetrates cell walls and destroys DNA, RNA and metabolic enzymes. After microalgae cells are treated with NaClO solution, the intracellular chlorophyll loses their activity, and the fluorescence intensity usually decreases exponentially with time. Therefore, the fluorescence intensity of microalgae is a direct indicator of their viability. We stipulated in this work that the chemical inactivation of microalgae was accomplished if the fluorescence intensity of microalgae cells reduced to less than 3% compared to their original intensity and maintained for at least 5 min. According to our experiments, this condition can be satisfied in about 20 min.

A concentration gradient generator was designed to quickly and accurately produce and screen the desired concentrations of NaClO for microalgae inactivation. Firstly, the diluent (deionized water, 0 ppm) and 250 ppm NaClO solution were injected into the concentration gradient generator using a syringe pump at a rate of 6 μL/min in each inlet, and the pumping process was stopped after 5 min. Thus, 10 μL of NaClO solution with concentrations of 0, 50, 100, 150, 200, and 250 ppm was produced in six sample wells, respectively. Then, the suspension of *Pyramimonas* sp. Was oscillated uniformly, and manually loaded into the six sample wells by a pipette (10 μL in each well, here the concentration of *Pyramimonas* sp. Is 240 cells/μL). The cell viability in each sample well was monitored for 20 min using the photon detector, and was displayed on a computer in real time by a LabVIEW program. The viability of microalgae cells was evaluated according to their relative change of fluorescence intensity as denoted in Equation (12):

(12) $$V_{algae}=(I_{1}-I_{background})/(I_{2}-I_{background}),$$

where *V_algae_* represents the relative cell viability, *I*_1_ represents the fluorescence intensity of the treated cells, *I_background_* denotes the system background fluorescence, and *I*_2_ represents the fluorescence intensity of the untreated cells.

**Figure 7:**
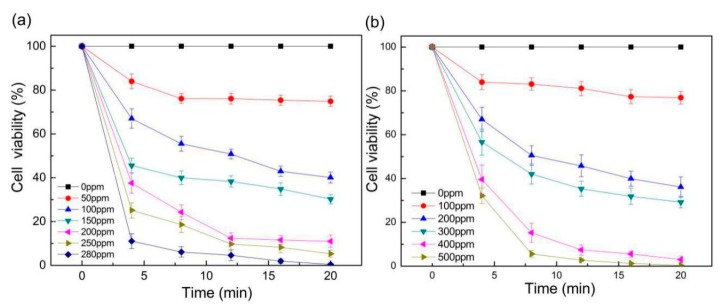
The change of relative viability of (**a**) *Pyramimonas* sp. and (**b**) *Chlorella* after treatment with NaClO under gradient concentrations for 20 min.

The viability of all *Pyramimonas* sp. Cells was normalized according to those treated with deionized water (0 ppm) as 100%. The change of microalgae viability after treatment with different NaClO concentrations is shown in Figure 7a. Typically, the viability of algae cells decreased rapidly in the first 8 min of treatment, and then gradually became stabilized. The cell viability also reduced more rapidly under higher NaClO concentrations. Under the highest NaClO concentration that of 250 ppm screened in this round, the cell viability was still about 8% after 20 min. It is to say, the NaCIO solution with a concentration of 250 ppm is insufficient. Therefore, we repeated the experiment process and adjusted the concentrations at the inlets of the concentration gradient generator and produced finer concentration gradient from 250 ppm to 300 ppm at six outlets. In this second round of screening, the viability of *Pyramimonas* sp. Reduced to almost zero within 20 min under the NaClO concentration of 280 ppm. Similarly, we screened the NaClO concentrations for inactivation of *Chlorella* (580 cells/μL) samples, as shown in Figure 7b. It was found that the *Chlorella* suspensions processed in this study required 500 ppm NaClO concentration for complete inactivation in 20 min. Thus, we realized rapid screening of inactivation chemicals to optimize the reagent concentration, as well as the treatment time for a particular microalgae species.

### 3.3. Comparison between the Fluorescence Method and Microscopic Method

**Figure 8:**
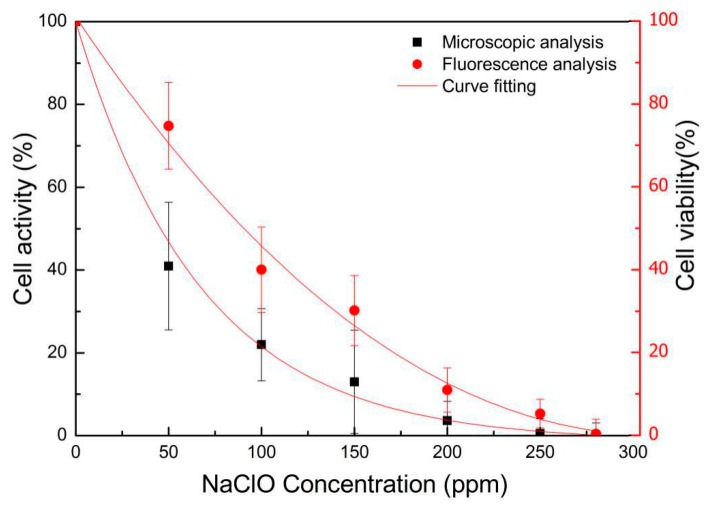
Comparison of the fluorescence detection method and the microscopic analysis (a case study of *Pyramimonas* sp. cells).

We also compared the conventional microscopic method with the presented fluorescence method for analyzing the viability of treated *Pyramimonas* sp. cells. The results in Figure 8 showed a general trend that the viability of *Pyramimonas* sp. decreased under various NaClO concentrations using both methods. However, the data obtained by the microscopic method exhibited very large error ranges and deviated considerably from those obtained by the fluorescence method. This is because the microscopic method is based on the observed physical morphology of cells, and induces subjective human errors in judging the activity of microalgae. On the other hand, the fluorescence method is based on the intracellular chlorophyll detected by a single-photon counter, which is very sensitive and eliminates human errors in the analysis. Therefore, the fluorescence detection module in this system provides unique advantages in terms of accuracy and reliability for the rapid analysis of microalgae.

## 4. Conclusions

Separation and analysis of microalgae species from ship ballast water are important to control maritime transportation induced pollutions and protect the marine ecological environment. Herein, a novel microfluidic system comprising microalgae cell separation, inactivation and viability analysis was demonstrated in this study. To the best of our knowledge, this is the first report to combine DLD separation, concentration gradient generator and chlorophyll fluorescence detection in a system for treatment and analysis of microalgae. This system was tested with two algae species: *Pyramimonas* sp. and *Chlorella*. It was shown that the system realized the rapid separation of these two species with separation efficiency up to 80% and a maximum throughput of 200 μL/min. It was found that the optimal separation efficiency for both species was achieved when the sheath flow rate was about 1/3 of the sample flow rate. After separation, the separated microalgae cells were introduced in the sample well array that contains different concentrations of the NaClO solution for viability study, while the cell viability was monitored in real time by a single-photon counter. The system proposed in this study has the major advantages of compact size, low cost, fast speed and automation, which is expected to contribute to the broad fields of ship ballast water treatment, microalgae management and marine ecological environment monitoring.

## Figures and Tables

**Table 1:** Solution concentrations at different levels.

	1	2	3	4	5	6
Level 1	0%	100%				
Level 2	0%	50%	100%			
Level 3	0%	40%	60%	100%		
Level 4	0%	30%	50%	70%	100%	
Level 5	0%	20%	40%	60%	80%	100%
